# Systematic Review of the Effects of Exercise and Physical Activity on the Gut Microbiome of Older Adults

**DOI:** 10.3390/nu14030674

**Published:** 2022-02-05

**Authors:** Catarina Ramos, Glenn R. Gibson, Gemma E. Walton, Daniele Magistro, Will Kinnear, Kirsty Hunter

**Affiliations:** 1 Sport, Health and Performance Enhancement (SHAPE) Research Centre, Department of Sport Science, Nottingham Trent University, Nottingham NG11 8NS, UK; Daniele.magistro@ntu.ac.uk (D.M.); William.kinnear@ntu.ac.uk (W.K.); Kirsty.hunter@ntu.ac.uk (K.H.); 2 Department of Food and Nutritional Sciences, The University of Reading, Whiteknights, Reading RG6 6AP, UK; g.r.gibson@reading.ac.uk (G.R.G.); g.e.walton@reading.ac.uk (G.E.W.)

**Keywords:** gut microbiome, older adults, healthy ageing, physical activity, exercise, gut microbiota, ageing, health

## Abstract

Recent evidence suggests that exercise/physical activity (PA) can beneficially alter the gut microbiome composition of young people, but little is known about its effects in older adults. The aim of this systematic review was to summarize results of human studies that have assessed the effects/associations of PA/exercise on the gut microbiome of older adults and to better understand whether this can help promote healthy ageing. Seven studies were included in the review and overall, exercise and increased amounts of PA were associated with decreases in the abundance of several well-known harmful taxa and increases in the abundance of health-promoting taxa. Altogether, the findings from the included studies suggest that exercise/PA have a beneficial impact on the gut health of older adults by improving the gut microbiome composition. However, due to methodological and sampling disparities, it was not possible to reach a consensus on which taxa were most affected by exercise or PA.

## 1. Introduction

In 2020, there were 727 million people aged over 65 worldwide [1]. Over the next 30 years, the number of people aged above 65 is expected to double, reaching around 1.5 billion people globally by 2050 [1]. The population is not only getting older but also more inactive with older adults being the age group that spends the most time sitting (65–80% of their waking time) [2] and being most prone to age and sedentary-related diseases. This is disturbing since physical inactivity is the fourth leading cause of death worldwide [3] and a primary cause of many chronic diseases [4], some of which are associated with ageing. It has also been suggested that physical activity (PA) may have similar effects when compared to drug interventions in terms of mortality benefits and secondary prevention of some cardiovascular diseases (CVDs) and diabetes [5]. Recent findings have shown that PA can improve the gut microbiome—the totality of the mixed community of microorganisms, including genetic components, microbial biodiversity, and their resulting functionality [6] which, in turn, might be beneficial for the process of healthy ageing.

The gut microbiome contains trillions of microorganisms, bacteria, archaea, viruses, protozoa, and fungi that are responsible for several essential functions related to host physiology, such as the digestion and absorption of nutrients, and production of metabolites that can affect whole-body metabolism, immune system, energy homeostasis, and inflammatory status [7]. The metabolites produced by the gut bacteria include: (i) Short-chain fatty acids (SCFAs), which can provide ~10% of daily energy requirements in humans [8], regulate glucose homeostasis and cholesterol metabolism [9], and modulate the immune system [10]. (ii) Bile acids (BAs), which are involved in the absorption of lipid-soluble vitamins, regulate triglycerides and help maintain the gut barrier function [11] as well as exert antimicrobial effects, depending on their type and concentration [12]. (iii) Lipopolysaccharide (LPS) or endotoxin, which is a glycolipid that is present in the outer membrane of Gram-negative bacteria (i.e., Proteobacteria) [13]. LPS is associated with insulin resistance [14] and intestinal homeostasis disruption, thus resulting in an increase in the permeability of the intestinal membrane which allows bacteria to translocate, consequently activating the immune system and inducing inflammation [15]. (iv) Vitamins. Some bacteria (40–65%) in the gut have the ability to synthesise several vitamins of the B family [16], as well as vitamin K [17,18]. Based on the above, it is understandable why the gut microbiome is important for metabolism and overall health. There is crosstalk and cross-regulation between several host organs and microbes in the gut [15,19,20], which then creates a homeostatic relationship between the gut and the host. This is why when there are defects in the gut microorganism population, it has whole-body repercussions which is, for example, what can happen during the ageing process.

The gut microbiome changes throughout the life of the host reaching maturation at 2–3 years old [12]. Afterwards, it remains relatively stable during adulthood and begins to change as the host starts ageing [21]. During the ageing process, the gut microbiome becomes unstable and presents a reduced diversity [22]. The loss of bacterial diversity in the aged gut has been associated with increased frailty and reduced cognitive performance [23] and was inversely correlated with physical function and institutionalization of older subjects [24]. Besides losing its diversity, it also loses its resilience [25] alongside changes in its composition. In terms of composition, an ageing gut microbiome is characterized by a reduced abundance of *Bifidobacterium* [26,27,28,29,30] and *Lactobacillus* [31], an increased abundance of Proteobacteria [26,32] and Bacteroidetes [33], a reduced number of SCFAs producers [25,34] including butyrate producers [26]. All of these compositional changes combined with physiological and lifestyle changes that occur during the ageing process such as reduction of PA/exercise [35], changes in taste sensation [36], decreased saliva production [22], weakened chewing strength [33,37], polypharmacy [38], reduced intestinal motility [39], and reduced production of mucin [40] will contribute to an increased predisposition to dysbiosis (an imbalance in the composition of the gut microbiome). Dysbiosis has been associated with several diseases and conditions that are prevalent in older adults, such as diabetes type 2 [41], inflammatory bowel diseases (IBD) [42], frailty [25], insulin resistance [43], atherosclerosis and stroke [44,45], dementia [46], hypertension [47], Alzheimer’s disease [48], Parkinson’s disease [49], colorectal cancer [50], rheumatoid arthritis [51], osteoporosis [52], cognitive decline [53], and increased barrier permeability and inflammatory cytokine expression [31,54].

There are several factors that negatively affect the composition of the gut microbiome (e.g., medications, lifestyle factors, and physiological factors) during the ageing process. That is why finding tools that can counteract these changes is of utmost importance in order to achieve healthy ageing.

PA/exercise might be a way to improve the gut microbiome during ageing since it influences several organs, cells, and tissues, and involves several molecular pathways. Previous studies have shown that PA/exercise have several benefits during the ageing process such as improvements in cognitive function [55,56], promotion of cardiovascular health [57] and improvements in the musculoskeletal system [58,59,60], making it a precious tool to counteract the age-associated changes that occur in those systems. Besides the benefits mentioned previously, recent studies [61,62,63,64] on the effects of exercise on the gut microbiome of older people have suggested that exercise might beneficially affect its composition and even reverse some age-associated taxonomical changes, ultimately leading towards healthy ageing. This is a relatively new topic of research. As such, human intervention studies are limited and there are only a few observational studies that have assessed this research question [61,63,64,65,66,67,68]. This systematic review aims to summarise results of human studies that assess the effects/associations of PA/exercise on the gut microbiome of older adults, to better understand whether physical activity can positively influence the gut microbiome of older adults and whether it can help promote healthy ageing. 

## 2. Materials and Methods

**Figure 1:**
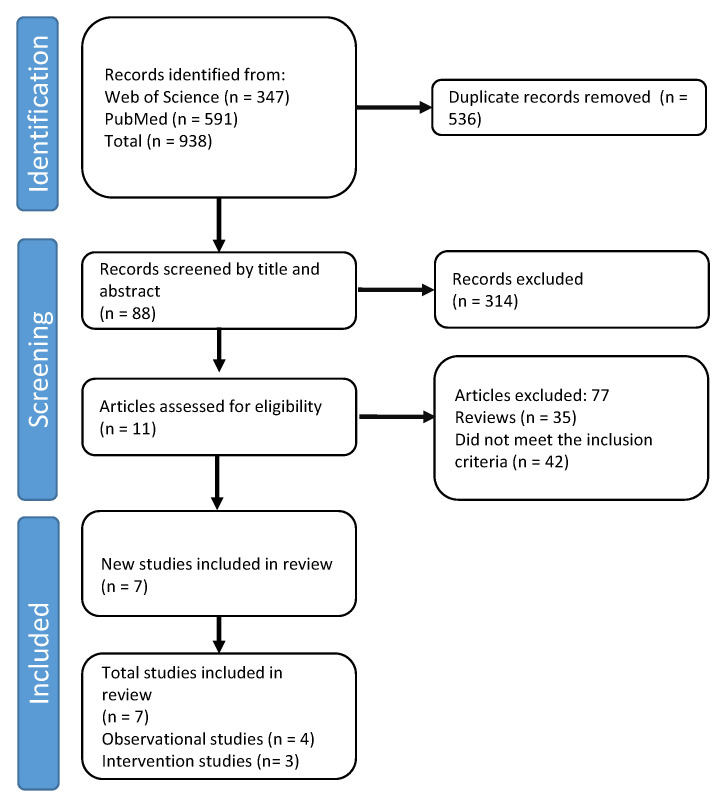
PRISMA flow diagram showing the study selection process.

The systematic review was performed according to the Preferred Reporting Items for Systematic Reviews and Meta-Analysis statement (PRISMA) [69] (Figure 1).

### 2.1. Literature Search

A literature search was carried out using PubMed and Web of Science between November 2020—November 2021 for all studies (not date restricted) published in English combining the terms “gut microbiome” OR “gut microbiota” AND “exercise” OR “physical activity” AND “ageing” OR “older adults” OR “elderly”. References lists in original papers and reviews were examined and a literature search was also conducted by following up references quoted on relevant articles.

### 2.2. Study Selection

Articles identified during the literature search were assessed using the PICOS search strategy and according to the inclusion criteria presented in Table 1. To be included, observational studies were required to focus on comparing the gut microbiome between older adults engaging in different amounts of physical activity. Intervention studies were included if they assessed the effect of any type of exercise intervention on the gut microbiome of older adults. Studies written in languages other than English, animal studies, editorials, commentaries, discussion papers, and conference abstracts were excluded (Table 1). The Newcastle Ottawa Scale (NOS) [70] was adapted and used to assess the risk of bias for cross-sectional studies and the Cochrane risk tool for randomised trials (RoB2) [71] was used to assess possible bias in randomised controlled trials. 

### 2.3. Study Characteristics

Of the seven studies that met the inclusion criteria, four were observational and three were randomised controlled trials (RCTs) which included an exercise intervention: two RCTs were conducted in Asia (China and Japan) and one in the United States, two observational studies used a population sample from America, one from Sweden and the other one from Ireland. Sample sizes of the intervention studies were relatively small, ranging from 12 to 33 individuals, whereas those from the observational studies were between 28 and 897 individuals.

#### Exclusion and Inclusion Criteria

The age for inclusion differed between studies, ranging from 50 to 98 years old. Two studies included Body Mass Index (BMI) ranges in their inclusion criteria (between 18.5–25 kg/m^2^ and 20–40 kg/m^2^ [61,68]), one only accepted subjects who had maintained a consistent lifestyle, diet, and body weight in the most recent decade [63], and two studies only included subjects with HbA1C < 6.5% and 7.5% [61,64], respectively. Additionally, fasting blood glucose < 7mmol·l^−1^, ability to live independently in the community and no diagnosis of type I or II diabetes were the inclusion criteria for the study performed by Zhong and colleagues [64]. Regarding exclusion criteria, only one study excluded subjects who consumed probiotics [66], two studies excluded subjects who took/had been taking antibiotics [66,68], three studies excluded subjects with tumours or malignancies [64,65,68] and two studies excluded subjects with gastro-intestinal (GI) diseases [63,65]. Regarding cardiovascular diseases (CVDs), only one study excluded subjects with different types of CVD and diagnosed with diabetes [64]. Moreover, Erlandson et al. [61] excluded participants who had taken sex hormone supplementation for the previous 3 months and intramuscular testosterone.

### 2.4. Methodology

#### 2.4.1. Observational Study Designs 

Four of the seven studies included in this review employed an observational study design [65,66,67,68]. One study assessed the associations between PA level and taxonomical composition of 373 older adults aged between 78–98 years old [66] and another study [68] used data from the American Gut Project to determine associations between PA/exercise and gut microbiome composition in older subjects. Additionally, one study [65] compared the taxonomical composition of 70 community dwelling older adults and 28 senior orienteers all aged above 65 years and another one [67] assessed associations between physical activity and gut bacteria in a population of 100 older adults aged between 55 and 74 years.

#### 2.4.2. Intervention Study Designs 

Three of the seven studies employed an exercise intervention [61,63,64]. Taniguchi et al. [63] ran a randomised crossover trial with 33 older adult men aged between 67–72 years old and employed a 5-week exercise intervention. Participants were randomly allocated to either the exercise group or the control group (no exercise) and after 5 weeks they switched conditions. The intervention consisted of exercising 3 times a week with intensity increasing from 60 to 75% VO_2peak_. Endurance exercise consisted of cycling on a cycle ergometer for 30 min during the first two weeks and then increasing to 45 min during subsequent weeks. Zhong et al. [64] conducted a randomised controlled trial with a between subject design comprising of 12 healthy sedentary women aged above 65 years. Participants were randomly allocated to either the combined exercise group or the control group. In the control group, participants were asked to not participate in exercise and were instructed to watch health related videos twice a month. For the exercise group, the intervention consisted of an 8-week exercise regime of aerobic combined with resistance exercise, consisting of 60 min per session, performed four times per week. Aerobic exercise consisted of chest extensions, claps, press and pulls, with each exercise lasting around 4 min with a resting period of 20 s between them. The resistance exercise was composed of six different exercises for the upper and lower body using an elastic band (chest expansion, arm curl, back stretch, shoulder crossing, side leg raiser, and push-kicks). It started with two sets of 8–10 reps further increasing to three sets of 12–15 reps. The study by Erlandson et al. [61] employed a randomised between subjects design with 15 participants (95% men) aged between 50–75 years; it included a 24 week aerobic and resistance exercise intervention consisting of three sessions per week. All participants completed three exercise sessions during the first 12 weeks. During the first 2 weeks, participants exercised at a low intensity (20–30 min of treadmill walking at 30–40% VO_2max_ combined with 3 × 8 reps at 40–50% 1-RM) increasing to 50 min by the end of the first 12 weeks and at an intensity ranging between 40–50% VO_2max_ combined with 60–70% 1-RM. On week 13, participants were randomised to either continue the moderate or start high intensity exercise (60–70% VO_2max_ and >80% 1-RM).

#### 2.4.3. Measurements

##### Physical Activity and Physical Function

Physical activity level was assessed using accelerometers [66,67], Frändin–Grimby Activity Scale (FGAS) [65] and IPAQ [64]. Only one study assessed physical function [64] using a chair-sit-and-reach test, grip strength, single-leg standing with eyes closed, and 30 s chair stand.

##### Diet

Nutrient intake was assessed using food frequency questionnaires (FFQ) [61,63,65,66,67] and a brief self-administered diet history questionnaire [63]. Two of the studies, one observational and one intervention, did not measure/take into account nutrient intake [64,68].

##### Blood Biomarkers

Only three studies took blood samples and the blood biomarkers analysed included HbAc1 [61,64], lipid profile [63,64], and fasted glucose [64].

##### Anthropometric Measures and Body Composition

Three studies [61,64,67] measured height and weight and then calculated BMI. Only one of the studies assessed body composition using magnetic resonance spectroscopy and imaging [63]. The other three did not assess body composition or take any kind of anthropometric measures.

##### Microbiome Quantification and Diversity Analysis

Six of the studies used 16s rRNA gene sequencing [61,63,64,66,67,68]; however, different regions were amplified: region V3-V4 [61,63,67] and V4 [64,66,68]. The other study used next-generation sequencing [65].

Alpha diversity (species diversity in a single microbial sample) was calculated in all of the studies using the Shannon Index (calculates diversity including richness and evenness) and some used additional measures, such as observed species [63,67] and Chao index (richness of microbial communities—number of bacterial species) [64,67]. Fart et al. (2020) additionally used Bray Curtis distances, Langsetmo et al. (2019) used inverse Simpson index, and Zhu et al. (2020) used QIIME diversity alpha-rarefaction visualizer. Two studies [61,64] used Sobs. Zhong et al. (2020) additionally used Ace indexes (richness of microbial communities—number of intestinal bacterial species). Six of the studies calculated Beta diversity. Estimates for β-diversity included principal coordinated analysis (PCoA) (used to study similarity or heterogeneity of a microbiota community composition) of weighted and unweighted Unifrac [63,64,65,66,67] and Bray–Curtis dissimilarity score [61,63,65].

## 3. Results

The main characteristics of the studies included in this review are presented in Table 2 and the synthesised findings from the included studies regarding taxonomical changes due to/associated with exercise/PA are presented in Table 3. Taxa belonging to five phyla were found to be statistically different when the studies were combined; Firmicutes had the most differences, followed by Proteobacteria, Bacteroides, Actinobacteria, and finally, Verrucomicrobia. Regarding changes at the phylum level, it was reported that exercise/PA increased the abundance of Actinobacteria [68] but decreased the abundance of Firmicutes [64]. At the family level an increase in Clostridiaceae and a decrease in Oxalobactereaceae [64] was reported and two studies [64,67] reported an increase in Lacnhospiraceae. Focusing on the Bacteroidaceae family, two studies presented contrasting results. Zhu et al. (2020) observed an increase in abundance of these whereas Zhong et al., (2020) observed a decrease in abundance caused by exercise. At the genus level, one study showed a decrease in *Adlercreutzia* and *Coprobaccillus* [66], two studies found decreased abundances of *Clostridioides* (formerly *Clostridium*) [63,64], and one study found a decreased abundance of *Streptococcus* [67] associated with PA/exercise. Fart et al. (2020) found a reduced abundance of both *Bilophila* and *Parasutterella* and Zhong et al. (2020) reported reduced abundance of *Escherichia* associated with exercise/PA. Erlandson et al. (2021) observed a decrease in *Succinivibrio*, *Prevotella*, and *Oribacterium* after the exercise intervention. In contrast, it was reported that increases in *Bifidobacterium* [61,67], *Ruminococcus*, *Prevotella*, and *Clostridioides* XI [67] were positively associated with/induced by PA/exercise. Zhong et al. (2020) demonstrated increases in *Roseburia*, *Akkermansia*, and *Mitsuokella* after the exercise intervention and Zhu et al. (2020) observed increases in *Paraprevotella*. Fart et al. (2020) observed an increase only in *Faecalibacterium* while two studies [61,63] observed increases in both *Oscillospira* and *Anaerostipes*.

Regarding alpha diversity, none of the studies found any changes due to exercise [61,63,64] or associated with PA/exercise [65,66,67]. The results for beta diversity differed between studies. While some studies found no differences [63,64] one found a weak relationships [66] and others found significant differences in this diversity index associated with/due to PA/exercise [61,65,67].

Some of the studies included found certain bacteria to be associated with several domains of physical function, physical activity behaviours, and cardiometabolic phenotypes. For example, Zhong and colleagues [67] demonstrated that standing time was positively correlated with the abundance of butyrate-producing and anti-inflammatory bacteria (e.g., Lachnospiraceae, *Bifidobacterium*, and Ruminococcaceae) and that moderate to vigorous PA was positively associated with the abundance of Lachnospiraceae and *Prevotella*. In contrast, it was found that sedentary time was associated with lower abundance of Ruminococcaceae and with higher abundance of *Streptococcus*. Additionally, Taniguchi et al. [63] found associations between cardiometabolic phenotypes and changes in *Oscillospira* and *C. dificille*. More specifically it was observed that changes in the abundance of *Oscillospira* were negatively correlated with changes in body fat percentage and HbA1c during the intervention and that this genus was positively correlated with changes in plasma HDL. Focusing on *C. difficile*, the abundance of this species was positively correlated with changes in visceral fat, systolic blood pressure, and HbA1c and negatively correlated with changes in VO_2peak_, total cholesterol, and plasma LDL. Moreover, Zhong and colleagues [64] found that certain bacteria were associated with different domains of physical function. More specifically, *Parabacteroides* were negatively associated with the single leg-standing test and the 30s chair stand test was positively associated with *Akkermansia*. In addition, the sit and reach test was positively associated with *Akkermansia, Roseburia, Mitsuokella*, and *Prevotella*. The handgrip test was positively associated with Lachnospiraceae, *Roseburia*, and *Mitsuokella* and negatively associated with *Bacteroides*.

## 4. Discussion

The three intervention studies included in this review found that an exercise protocol resulted in significant changes in taxonomical abundances compared to pre-intervention values. More detailed analysis suggests that PA/exercise has a positive effect on the composition of the gut microbiome of older adults. Taniguchi et al. (2018) found that after a 5-week aerobic exercise intervention in older men there was a significant increase in relative abundance of the genus *Oscillospira* and a decrease in *Clostridioides* *difficile*. Interestingly, the genus *Oscillospira* was previously found to be negatively associated with metabolic disturbances but positively associated with leanness and reduced BMI [84,85] which could explain why this genus might be increased after an exercise intervention. In contrast, *Clostridioides* *difficile* is a well-known pathogen that has been associated with increased inflammation and has been shown to be capable of altering the gut microbiome towards a more pathogenic composition [87], so the decrease in this species induced by exercise can be a mechanism by which it improves overall composition of the gut microbiome in older adults. Zhong et al. (2020) found that after an 8-week combined exercise intervention in older woman there was a decrease in abundance of the Firmicutes phylum, Bacteroidaceae family, and the genera *Clostridioides* and *Escherichia*. In contrast, increases in the Lachnospiraceae family and in the genera *Roseburia*, *Mitsuokella* and *Akkermansia* occurred. The family Bacteroidaceae has been previously negatively associated with body weight and fasted plasma insulin [95], however, there are several discordances in the literature and it appears that members of this genus can be both beneficial and pathogenic depending on several factors such as the taxonomical composition, diet or whether saccharolytic or proteolytic species are considered. The genera *Clostridioides* (which includes *C. difficile*) and *Escherichia* are well-known to include potential pathogens that have been associated with inflammation and several GI tract diseases [87,107]. Moreover, they have been shown to be increased during ageing. In contrast, members of the Lachnospiraceae family (which includes the genus *Roseburia*) have been shown to reduce inflammation and are SCFA producers [88,111]. Additionally, the genus *Roseburia* was found to decrease concomitantly with muscle mass loss and dysfunction [89] thereby highlighting a possible link between muscle mass and gut health during the ageing process.

Regarding *Akkermansia*, this genus has been positively associated with overall health, increased insulin sensitivity, and glucose tolerance [109], and weight loss, and has been negatively associated with insulin resistance, dyslipidemia and BMI [112,113]. Focusing now on older adults, lower amounts of *Akkermansia* were associated with poorer sleep quality [114] and higher amounts were positively associated with attention, memory and executive function [115]. Additionally, supplementation with *Akkermansia municiphila* was shown to reduce total plasma cholesterol and fat mass in overweight and obese adults [110]. This species also appears to be responsive to exercise as its abundance was increased after 6-weeks of endurance exercise in overweight sedentary women [116]. Altogether, *Akkermansia* seems to be a genus with several important functions in terms of metabolism regulation and health promotion and it has been identified as a possible next-generation probiotic due to these features. If PA/exercise are able to increase the amounts of *Akkermansia* in the gut, this might be another way by which exercise might promote healthy ageing. Finally, members of the genus *Mitsuokella* are butyrate producers [93] and an increase in butyrate producers due to exercise aligns with previous studies that found that athletes had higher SCFA producing bacteria [117,118] when compared to sedentary controls, therefore suggesting that exercise can increase SCFA production. 

Erlandson et al. (2021) found that after a 24-week of combined exercise intervention with a gradual increase in intensity in sedentary subjects (95% men), there was an increase in the genera *Bifidobacterium*, *Oscillopsira* and *Anareostipes* and a decrease in *Oribacterium* and *Prevotella*. While physiological information about the genus *Anaerostipes* is limited, they are known to be acetate and butyrate producers [90] and have been found to have a beneficial role in renal function [91]. As such, based on the limited information available, it appears to be a health-related genus that is also responsive to exercise. The same situation is true for the genus *Oribacterium*. Although there is limited information about this genus in the literature, it was previously found to be present in higher proportions in obese subjects [92] suggesting that it might be associated with obesity or metabolic dysfunction. The fact that exercise might reduce abundance of this genus could also be another mechanism by which it promotes health. Combining findings from the intervention studies, exercise was capable of changing the composition of the gut microbiome towards a healthier one in older people, by increasing abundances of bacteria associated with benefits and potential for SCFA production but decreasing the abundance of potential pathogens; this could ultimately counteract some of the negative effects of ageing on the gut microbiome, therefore leading to better overall health. However, due to the fact that exercise protocols differed substantially between studies, it is difficult to compare them and find a common effect of exercise on the gut microbiome composition since different types/modes of exercise produce different physiological and molecular responses.

Observational studies demonstrated that higher levels of PA/exercise were associated with decreases in the relative abundance of the Oxalobacteriaceae family and the genera *Coprobaccillus*, *Adlercreutzia*, *Bilophila* and *Streptococcus* and increases in the abundance of phylum Actinobacteria, the families Clostridiaceae, Bacteroidaceae, Lachnospiraceae and the genera *Prevotella, Clostridioides* XI, *Ruminococcus, Paraprevotella*, *Bifidobacterium* and *Faecalibacterium*. The family Oxalobacteroidaceae has been shown to have increased abundance in patients with cholangiocarcinoma [102] and knee osteoarthritis [103], so an exercise-induced reduction of this family may be positive for health. The genus *Coprobaccillus* has previously been shown to be positively correlated with frailty and biological ageing [78] whereas *Adlercreutzia* was detected in high abundance in patients with back pain and positively correlated with BMI and inflammation [73]. Additionally, members of the genus *Bilophila* produce hydrogen sulphide, which is known to have cytotoxic effects on colonocytes and inhibits butyrate production [105]. Higher concentrations of hydrogen sulphide have been shown to induce inflammation [106]. Finally, members of the genus *Streptococcus* are well-known pathogens since they are involved in the development of metabolic disorders, diabetes, colon cancer and have pro-inflammatory effects [94]. In summary, in these observational studies, increased PA/exercise was associated with decreases in the relative abundance of potentially pathogenic taxa suggesting that older people who engage in more PA/exercise tend to have a healthier gut microbiome when compared to their sedentary counterparts, therefore feasibly improving overall health.

Regarding the taxa shown to be positively associated with an increased level of PA/exercise, the Actinobacteria phylum (which contains the genus *Bifidobacterium*) has been reported to have positive effects on gut health such as inhibition of pathogens through the modulation of intestinal and immune responses [74], vitamin production [75], LPS reduction [77] and anti-inflammatory properties [76]. Members of the genus *Prevotella* are well-known SCFAs producers [97] and previous studies have found that higher amounts of exercise correlated positively with this genus [119]. However, there are contrasting results in the literature and some studies have shown that this genus might be associated with inflammation [96], consequently more research is needed in order to clarify the effects of *Prevotella* on the gut environment. The same applies to the *Ruminoccocus* genus; while some studies indicate that they are butyrate producers, others show that some species might be associated with depression whereas others might have anti-depressive effects [79]. Members of the *Paraprevotella* genus have been shown to be in reduced abundance in low functioning older adults [98] and sedentary women [99] with reduced abundance of members of this genus being associated with several diseases [100,101]. Finally, *Faecalibacteirum prauznitsii* has been positively associated with overall health. It has been shown to have anti-inflammatory properties through the secretion of some metabolites that are able to block the inflammatory cascade [81], it is a butyrate producer [82], and may offer protection against colon cancer [83]. Additionally, this genus is also responsive to PA/exercise since it was previously found that its abundance was increased after a 6-week exercise intervention [120] and that active women had higher proportions of this species when compared to inactive women [99]. Overall, findings from observational studies indicate that older people that are more active/do more exercise, tend to have increased abundances of health-related bacteria and decreased abundances of bacteria that are associated with disease or inflammation. 

**Figure 2:**
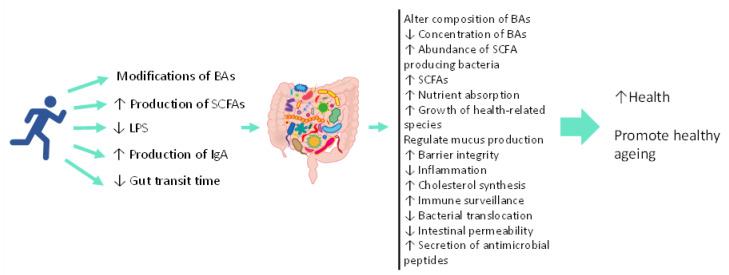
The mechanisms by which PA/exercise might promote healthy ageing via gut microbiome alterations. ↑: Increase; ↓: Decrease; BAs: Bile acids; SCFAs: Short chain fatty acids; LPS: lipopolysaccharide; IgA: Immunoglobulin A.

Altogether, results from the intervention and observational studies support the beneficial role of PA/exercise on the gut microbiome of older adults and therefore the potential to promote healthy ageing. Physical activity/exercise may act through changes in the gut environment such as increases in the production of SCFAs, modification of bile acids, reduction of LPS, reduction of transit time, regulation of mucus production which may, in turn, change the composition of the gut microbiome towards a healthier one by promoting the growth of health-related bacteria, and a decrease in harmful bacteria. These changes could lead to improved health and the promotion of healthy ageing (Figure 2). However, it should be noted that findings from the different studies vary and it is difficult to find a common conclusion or consensus with regards to which taxa are most responsive to exercise/PA in an elderly population as well as what types of exercise are most effective for improving the gut microbiota. This is in part due to the use of different methodological approaches for assessing microbial composition. Firstly, studies used different faecal collection procedures, DNA extraction kits and DNA sequencing techniques. Different stool samplings and DNA isolation kits can affect DNA quality and bacterial composition [121]. Secondly, the use of different hypervariable regions has been previously shown to produce different results [122]. Thirdly, the use of different programs or databases to identify different OTUs and taxonomical changes can affect estimation of relative bacterial abundance [123]. Guidelines should be created in this field with regards to the use of statistics and standardised procedures to collect, extract and analyse bacterial composition to standardise results, allowing comparisons between studies.

### Suggestions for Future Studies

This is an expanding research field and more is needed to investigate the effects of different types of exercise on the gut microbiome of older people coupled with mechanistic studies to elucidate cellular and molecular mechanisms behind interactions between exercise and the gut. Additionally, most studies, to date, that investigate the relationship between exercise/PA with the gut microbiome are observational and performed in Asian populations, therefore more studies are needed in western populations. Additionally, more intervention studies should be performed in order to assess the effects of several types of exercise on the gut microbiome composition of older adults. Moreover, studies should try to assess all parameters that might influence the gut microbiome composition (e.g. body composition, diet, smoking status, intake of pre-probiotics, etc) to reduce confounding effects. Ultimately it will be important to determine the best type of exercise to improve taxonomical and functional composition of an aged gut to promote optimal ageing. Coupled to this, more studies are needed to define what a healthy gut microbiota might look like in aged individuals. Finally, it is important to assess how long the effects of exercise on the gut microbiome might last in older people after exercise cessation and how long it takes for exercise to alter taxonomic profiles.

## 5. Conclusions

There is limited research on the effects of exercise/PA on the gut microbiome of older people and the few intervention studies available have small sample sizes. Overall, findings from the included intervention and observational studies indicate that exercise/PA can have a beneficial impact on the gut microbial composition of older adults, however, due to methodological and sampling disparities, it was not possible to reach a consensus on which taxa is most responsive/influenced by exercise or physical activity in this specific population.

## Figures and Tables

**Table 1:** PICOS criteria for inclusion and exclusion of studies.

Parameter	Inclusion Criteria
Population	Older adults
Intervention	Measurement before and after an exercise or physical activity intervention
Comparison	Sedentary vs physically active Different exercise interventions
Outcomes	Taxonomical composition Bacterial abundance Alpha and beta diversities
Study type	Observational studies Randomised controlled trials

**Table 2:** Main characteristics of the studies included in this review.

Authors	N	Exercise Protocol	Measurements
[63]	33 elderly Japanese men (67–72 years)	Randomized crossover tria l5-week endurance exercise, 3 times/week The intensity increased from 60 to 75% VO_2peak_	16s rRNA (V3-V4) CRF test Brief self-administered diet history questionnaire MRI Lipid profile, HbAC1 Blood pressure Cardio-ankle vascular index
[66]	373 men between 78–98 years	Observational study	Accelerometer 16s rRNA (V4) FFQ
[65]	70 community-dwelling older adults + 28 physically active senior volunteers (orienteers) aged > 65 years	Observational study	Gastrointestinal symptom rating scale (GSRS) Hospital anxiety and depression scale (HADS) FFQ Franding–Grimby activity scale (assess PA level) Next-generation sequencing (NGS)
[68]	897 subjects aged > 60 years	Observational study. Detected the effect of exercise on the gut microbiota in elderly individuals by using the data from the American Gut Project	Used the data obtained from the American Gut Project 16s rRNA (V4)
[64]	12 physically inactive older women aged > 60 years	Randomized controlled trial 8-week exercise training of aerobic and resistance exercise 60’ sessions 4x/week of aerobic + resistance exercise	IPAQ Mini-mental state examination (MMSE) Health examination Height, body weight flexibility, strength, and balance Fasted glucose, total cholesterol. LPL. HDL HBA1c 16s rRNA (V4)
[67]	100 subjects 55–74 years	Observational study	Accelerometer BMI 16s rRNA (V3-V4) FFQ
[61]	15 participants (95% men) aged 50–75 years	Randomised Trial 24-week aerobic + resistance exercise intervention 2 weeks: low intensity (20–30′ treadmill walking at 30–40% VO2max + 3 × 8 reps at 40–50% 1-RM) increasing to 50′ by the end of 12 weeks at 40–50% VO2max + 60–70% 1-RM After 13 weeks: randomized to continue moderate or high intensity exercise (60–70% VO2max + >80% 1-RM)	VO_2max_ 16s rRNA (V3-V4) Gas chromatography to assess stool SCFA 3-day dietary survey

**Table 3:** Microbial changes observed in the included studies due to/associated with exercise/physical activity (adapted from [72]).

Phylum	Family	Genus	Physiological Effects/Associations Previously Detected
Actinobacteria ↑ [68]	Eggerthellaceae	Adlercreutzia ↓ [66]	Higher abundance in patients with back pain and positively correlated with BMI and inflammation [73]
	Bifidobacteriaceae	Bifidobacterium ↑ [67] ↑ [61]	Inhibit pathogens [74] Modulation of intestinal and systemic immune responses [74] Vitamin production [75] ↓ Inflammation [76], ↓ LPS [77]
Firmicutes ↓ [64]	Erysipelotrichaceae	Coprobacillus ↓ [66]	Positive correlation with frailty and associated with biological ageing [78]
	Ruminococcaceae	Ruminococcus ↑ [67]	Degrade and convert complex polysaccharides into a variety of nutrientsButyrate producers This genus has been associated with depression in some studies, although some species have anti-depressive effects [79]
		Faecalibacterium ↑ [65]	↓ Inflammation [80,81] Butyrate production [82] Protect against colon cancer [83]
		Oscillospira ↑ [63] ↑ [61]	Negatively associated with metabolic disturbances [84] Associated with leanness and ↓BMI [84,85]
	Clostridiaceae ↑ [68]	Clostridioides XI ↑ [67]	Increased in autistic children [86]
	Peptostreptococcaceae	Clostridioides ↓ [63] (C. difficile) ↓ [64]	Genus with several well-known pathogens (e.g. C. difficile) ↑ Inflammation [87] Alteration of gut microbiota composition [87]
	Lachnospiraceae ↑ [64,67]	Roseburia ↑ [64]	SCFAs production [88] Anti-inflammatory effects Abundance decreases concomitantly with muscle mass and dysfunction [89]
		Anaerostipes ↑ [61]	Production of acetate and butyrate [90] Beneficial role on renal function [91]
		Oribacterium ↓ [61]	Higher proportions were found in obese subjects [92]
	Selenomonadaceae	Mitsuokella ↑ [64]	Butyrate production [93]
	Streptococcaceae	Streptococcus ↓ [67]	Involved in the development of metabolic disorders, diabetes and colon cancer. Increases inflammation [94]
Bacteroidetes	Bacteroidaceae ↑ [68] ↓ [64]		Negatively correlated with body weight and fasted plasma insulin [95]. Members of this genus can be beneficial for the host and can be pathogenic as well, depending on several factors such as the taxonomical composition, geographic location or diet.
	Prevotellaceae	Prevotella ↑ [67] ↓ [61]	Beneficial for the GI tract and human health Propionate producersSome studies found that specific strains from the genus might be involved in inflammation [96]
		Paraprevotella ↑ [68]	Succinate and acetate producers [97] Reduced abundance in low functioning older adults [98] and sedentary women [99] Reduced abundance is associated with several diseases [100,101]
Proteobacteria	Oxalobactereaceae ↓ [68]		Increased abundance in cholangiocarcinoma patients [102] Increased abundance in patients with knee osteoarthritis [103]
	Sutterellaceae	Parasutterella ↓ [65]	Associated with IBS genesis and development [104] Associated with obesity, diabetes and fatty liver disease [65]
	Desulfovribionaceae	Bilophila ↓ [65]	Produces H_2_S which has cytotoxic effects on the gut membrane and inhibits butyrate production [105] Higher abundance induces systemic inflammation [106]
	Enterobacteriaceae	Escherichia ↓ [64]	This family has been associated with IBD and other GI tract diseases [107] LPS producers Pro-inflammatory
	Succinivibrionaceae	Succinivibrio ↓ [61]	Fiber degradation and typical in diets with high fibre and complex carbohydrates [108]
Verrucomicrobia	Akkermansiaceae	Akkermansia ↑ [64]	Decreased abundance was correlated with increased BMI [109] ↑ Insulin sensitivity and glucose tolerance [109] Reduces total cholesterol [110] Decreases fat mass [110]

We only included taxonomical changes that were statistically significant, ↑ Increased after or positively associated with exercise/physical activity, ↓ Decreased after or negatively associated with exercise/physical activity, METs—Metabolic syndrome; IBD (inflammatory bowel disease); LPS (lipopolysaccharide); UC (ulcerative colitis); GI (gastrointestinal); SCFAs (short-chain fatty acids); NAFDL (non-alcoholic fatty liver disease); TLR4 (toll-like receptor 4); BMI (body mass index); TG (triglycerides).
